# Urban–Rural Disparities in Life Satisfaction Among Older Koreans: Determinants and Healthcare Policy Implications

**DOI:** 10.3390/healthcare13111252

**Published:** 2025-05-26

**Authors:** Hyun-Chool Lee, Alexandre Repkine, Luwen Zhang

**Affiliations:** 1 Political Science Department, Konkuk University, Seoul 05029, Republic of Korea; lhc0609@konkuk.ac.kr; 2 Economics Department, Konkuk University, Seoul 05029, Republic of Korea; 3 Institute of Economics, School of Social Sciences, Tsinghua University, Beijing 100084, China; luwen-zhang@mail.tsinghua.edu.cn

**Keywords:** healthy aging, South Korea, instrumental variables estimation, determinants of life satisfaction

## Abstract

Background: This study examines how social and geographical factors shape life satisfaction among older adults in South Korea, which became a super-aged society in 2024. As Korea moves toward implementing a nationwide integrated eldercare system by 2026, understanding the contextual determinants of wellbeing among older populations is critical for effective policy design. Methods: We use nationally representative survey data and apply a two-stage instrumental variable regression approach to address potential endogeneity in estimating the effects of key predictors on life satisfaction. Results: Subjective health and social connectedness are positively associated with life satisfaction. Physical activity shows context-specific effects, with notable differences between urban and rural areas. Surprisingly, greater accessibility to government services is linked to lower levels of physical activity, indicating a possible unintended consequence of well-intentioned policy measures. Gender differences are also evident: in urban areas, women report higher life satisfaction than men, whereas in rural areas, men report higher satisfaction than women. Conclusions: These findings highlight the need for aging policies that are context-sensitive and socially inclusive. Policymakers should consider regional and gender-specific dynamics when designing programs to improve life satisfaction among older adults in a rapidly aging society.

## 1. Introduction

As South Korea became a super-aged society in 2024, with over 20% of its population aged 65 and older (Lee [1]; Repkine and Lee [2]), identifying the determinants of healthy aging has become increasingly important. The growing demand for healthcare and social services (Giri et al. [3]) calls for policies that promote older adults’ engagement in economic and social life—an outcome closely tied to their life satisfaction, which significantly impacts health (Boccaccio et al. [4], and Baeriswyl and Oris [5]).

Numerous studies highlight the close link between life satisfaction and health among older adults. For example, research from Canada [6], South Africa [7], and India [8] shows that life satisfaction is strongly associated with better mental and physical health and reduced mortality. Kim et al. [9] found that individuals with higher life satisfaction reported better physical health outcomes. In Korea, Lim et al. [10] and Gu et al. [11] find consistent evidence that higher life satisfaction among older adults correlates with improved health outcomes.

Given its importance for wellbeing, identifying the key drivers of life satisfaction is essential (Lee and Repkine [12]). While prior research has explored its determinants, the role of geographic context, and especially the urban–rural differences, remains underexplored. Even studies that consider such differences (e.g., Rey-Beiro and Martinez-Roget [13]; Lim et al. [10]) often overlook their statistical significance, leaving a gap in understanding location-based disparities. In this study, we statistically account for the disparity between rural and urban areas by including a rural–urban dummy variable into our model.

Understanding the urban–rural distinction is crucial in Korea, where 82% of the population is urbanized, but nearly a third of older adults still live in rural areas. These groups may experience social and environmental factors differently, affecting their life satisfaction. Rural seniors often face limited healthcare access (Chen et al. [14]), while urban areas offer more formal social opportunities (Sok et al. [15]), yet stronger social cohesion in rural communities may partly offset these disparities.

As Korea becomes a super-aged society, understanding urban–rural differences in senior life satisfaction is vital for effective policy. Urban seniors benefit from services but often face high costs and isolation, while rural seniors enjoy strong community ties but lack healthcare access and economic stability. These contrasts call for targeted policies to ensure equitable support. Encouraging older adults to move to rural areas with proper incentives could enhance wellbeing and help address regional depopulation, supporting both senior welfare and balanced development.

A key methodological challenge in studying life satisfaction is endogeneity—when determinants like social connections and perceived health may both influence and be influenced by life satisfaction. Ignoring this bidirectionality can bias estimates. This concern is especially relevant in the urban–rural context, where unobserved factors like lifestyle or policy differences may affect both outcomes and predictors.

This study uses a two-stage instrumental variables regression to examine the determinants of life satisfaction among older Koreans, focusing on urban–rural disparities and endogeneity. We hope that our study provides insights that can help policymakers design effective, location-specific strategies to enhance the wellbeing of Korea’s aging population.

This paper is structured as follows. Section 2 describes the survey data used in our study and the analytical framework employed, specifically the two-stage least squares approach, which addresses potential endogeneity issues in the regressors. Section 3 presents summary statistics and the regression results. Section 4 discusses empirical findings, while Section 5 concludes with a summary and policy implications.

## 2. Materials and Methods

### 2.1. Survey Data Acquisition Process

Our empirical analysis employs the National Survey of Older Koreans [16], which was conducted by the Korea Institute for Health and Social Affairs (KIHASA) in 2023. This survey, approved by Statistics Korea (Approval No. 117071, 18 August 2023), was carried out under the Elderly Welfare Act and covers 10,078 South Koreans aged 65 and older. The survey questions were reviewed by KIHASA’s Institutional Review Board (IRB), which granted approval on 28 July 2023 (IRB No. 2023-078). Data collection involved tablet-assisted personal interviews conducted with older individuals or their designated representatives.

We used R software (version 4.4.2, package ‘survey’) to detect outliers, identify non-sampling errors, and flag invalid responses, which were removed. To ensure sample representativeness, the survey employed a stratified random sampling approach. Population strata were based on the Korean Population and Housing Census conducted by the Korean National Statistical Office (NSO). Each of Korea’s seventeen regions was proportionally represented, with sample sizes determined using the adjusted square-root two-stage allocation method described in Schafer [17].

### 2.2. Variables and Measurements

#### 2.2.1. Life Satisfaction and Endogenous Regressors

Our dependent variable is life satisfaction, measured on a 1–5 Likert scale. Life satisfaction is linked to factors like social connectedness, physical activity, and health perception. Thus, Monteiro et al. [18] highlight the importance of social networks and community engagement for older adults’ life satisfaction, while Bavarsad and Stephens [19] emphasize the role of social connections in maintaining a sense of purpose.

Regular physical activity is strongly linked to higher life satisfaction as argued by Toros et al. [20], who found that elderly men who exercised reported greater life satisfaction, self-esteem, and self-efficacy. Similarly, An et al. [21] showed that physical activity correlates with improved life satisfaction in older adults. Qazi et al. [22] found that subjective health perception is also closely linked to life satisfaction in aging women.

Research suggests, however, a bidirectional relationship between life satisfaction and the three factors. Thus, Sakellariou [23] notes that strong social ties enhance mental wellbeing, and healthier individuals are more likely to form social connections. Wypych-Slusarska [24] shows that higher life satisfaction motivates physical activity, indicating reciprocity. Similarly, Mathentamo et al. [25] find a reciprocal link between life satisfaction and perceived health, where each influences the other over time.

The discussion above highlights the need to address endogeneity in analyzing the impact of social connectedness, physical activity, and subjective health perception on life satisfaction. In the next section, we describe the instrumental variables used in a 2SLS framework to correct for endogeneity biases and improve causal effect estimates, providing references to the existing literature to corroborate our choice of instruments.

#### 2.2.2. Instrumenting the Endogenous Determinants of Life Satisfaction

Instruments are variables correlated with endogenous regressors, such as social connectedness, but not directly with life satisfaction. We identified three sets of instruments for the potentially endogenous regressors, with a rural–urban dummy common to all. This highlights the role of the dwelling environment in life satisfaction through social connections, physical activity, and health perceptions. As argued by Repke and Ipsen [26], rural residents often report higher social participation than urban dwellers, while Cohen et al. [27] find that rural residents engage in more household physical activity, while urban dwellers participate in higher-intensity activities due to better access to sports facilities. Regarding self-reported health, Saha et al. [28] identify significant differences between rural and urban residents.

In addition to the rural–urban dummy, our instruments for social connectedness include the ‘has religion’ dummy, ‘can use Internet’ dummy, ‘ease of adaptation to informational changes’ Likert variable, and a dummy for having close friends nearby. Studies like Lim and Putnam [29] highlight that religious affiliation can enhance life satisfaction through congregation-based networks. Caton et al. [30] and Antonucci and Manalel [31] find that internet use and adaptability to informational changes influence social interactions. Tomini et al. [32] show that proximity to close friends enhances social support and connectedness.

For physical activity, we include indicators of infrastructure quality and government service accessibility along with the rural–urban dummy. Both indicators are sums of dummies representing various infrastructure and service dimensions, normalized between zero and one. The infrastructure indicator aggregates satisfaction with social overhead capital, accessibility to shops and medical facilities, and availability of transportation, roads, and green areas. The government services indicator includes accessibility to agencies, use of government-provided restaurants for seniors, and food delivery services. Our choice of indicators is supported by research, such as Jiang et al. [33], which finds that well-developed infrastructure like accessible street networks increases physical activity in older populations. Similarly, Sobczyk et al. [34] establish a positive link between physical activity and government service accessibility for older citizens.

Finally, we instrument subjective perception of one’s health with the smoking dummy and a frequency variable capturing alcohol consumption, again in addition to the rural–urban dummy variable.

#### 2.2.3. Exogenous Controls

Exogenous controls influencing life satisfaction include age, gender, income level, years worked, presence of family conflicts, and two variables measuring satisfaction with dwelling conditions and crime incidence.

The determinants above are widely examined in studies on older adults’ life satisfaction. For instance, Celik et al. [35] find that marital status, education, and income-generating work positively affect life satisfaction in Turkiye. Jeong [36] reports similar results for Korea’s elderly. Park et al. [37] discuss the roles of homeownership and family conflicts, while Cohen [38] shows that crime rates and perceived neighborhood safety significantly impact life satisfaction.

### 2.3. Statistical Analysis

#### 2.3.1. Two-Stage Least Squares

As argued above, social connections, physical activity, and subjective perception of health may be endogenous regressors with respect to the life satisfaction variable in the sense that causality may run in both directions. If this endogeneity problem is not addressed, the resulting coefficient estimates may be biased and inconsistent. A standard way of dealing with endogenous regressors is the two-stage least squares (2SLS) approach, a statistical method used to address endogeneity issues in regression analysis, where some explanatory variables are correlated with the error term, leading to biased estimates.

In 2SLS, the first stage involves predicting the problematic variables using instrumental variables that are correlated with them but not with the error term. In the second stage, these predicted values are used in place of the original variables to ensure unbiased and more reliable estimates. This method helps correct for endogeneity and improves the accuracy of regression results.

Formally, denote *Y* to be the dependent variable in the second-stage (main) regression, i.e., the life-satisfaction Likert variable. Let $\vec{X}$ be a vector of exogenous controls discussed above. Vector $\vec{Z}$ consists of the instrumental variables for the elements of $\vec{W}$, a vector of endogenous regressors.

The two-stage least squares estimation (2SLS) proceeds as follows:

First stage: estimate the reduced-form equations by regressing each endogenous regressor in $\vec{W}$ on a set of exogenous controls $\vec{X}$ and a set of instruments $\vec{Z}$:

(1) $$\vec{W}=\Pi_{X}\vec{X}+\Pi_{Z}\vec{Z}+\vec{u}$$

where $\Pi_{X}$ and $\Pi_{Z}$ are coefficient matrices for vectors $\vec{X}$ and $\vec{Z}$, respectively, and $\vec{u}$ is a vector of error terms with mean zero and finite variance, potentially heteroskedastic. Specification (1) is estimated by ordinary least squares (OLS) in order to obtain the predicted values $\vec{\hat{W}}$. To account for potential heteroskedasticity in the first-stage errors $\vec{u}$, we compute robust standard errors for the first-stage regression.

Second stage: regress the dependent variable *Y* on the predicted values of the endogenous regressors $\vec{\hat{W}}$ obtained in the first stage by estimating (1), and a vector of exogenous controls $\vec{X}$:

(2) $$Y={\vec{\beta }}_{X}\vec{X}+{\vec{\beta }}_{W}\vec{\hat{W}}+\varepsilon$$

where ε is the error term with a zero mean, finite variance, and possible heteroskedasticity. To account for potential heteroskedasticity in the second-stage errors ε, we compute heteroskedasticity-robust standard errors for the final 2SLS estimates. The 2SLS estimator will yield consistent estimates of ${\vec{\beta }}_{X}$ and ${\vec{\beta }}_{W}$, mitigating the bias caused by endogeneity in $\vec{W}$. We implement the 2SLS estimation of (1) and (2) using Stata 18. A detailed discussion of the 2SLS procedure can be found in Wooldridge [39].

#### 2.3.2. Testing for Regressors’ Endogeneity

To test whether social connections, physical activity, and subjective health are endogenous to life satisfaction, we use a version of the Hausman [40] test. Specifically, we test whether predicted residuals from the first-stage instrumented regressions in (1) have any explanatory power in a regression of life satisfaction on the potentially endogenous regressors and the exogenous controls. In other words, estimate the following regression where $\hat{\vec{u}}$ is a vector of predicted residuals from the first-stage regression (1):

(3) $$Y={\vec{\beta }}_{X}\vec{X}+{\vec{\beta }}_{W}\vec{W}+\vec{\rho }\hat{\vec{u}}+\varepsilon$$

We then test the null hypothesis $H_{0}:\vec{\rho }=\vec{0}$, which corresponds to the exogeneity of the potentially endogenous regressors $\vec{W}$, by applying a standard *F*-test to the joint significance of the elements of $\vec{\rho }$.

#### 2.3.3. Robustness and Sensitivity Analysis

In the course of our empirical work, we experimented with various ways to formally represent the concepts employed in our model. For example, one key determinant of life satisfaction—social connections—is captured through a construct comprising nine variables that reflect older Koreans’ involvement in educational activities, hobby clubs, frequency of contact with children, and similar forms of social engagement. While we explored different subsets of the fifteen available variables that could, in principle, represent social connections, these compositional adjustments led only to minor quantitative changes in the estimated coefficients, leaving qualitative results—such as the sign and statistical significance—largely unaffected.

## 3. Results

### 3.1. Summary Statistics

Table 1 presents summary statistics for life satisfaction—the dependent variable *Y* in specification (2)—and for the vector $\vec{W}$ of three endogenous regressors: social connectedness, physical activity, and subjective perception of health. Half of the respondents reported a social connectedness level below 0.4 and a physical activity level of 0.3 on a scale from 0 to 1, suggesting considerable scope for improvement in these areas. The distribution of life satisfaction scores is relatively symmetric, centered around a median of 3 on a scale from 1 to 5. A similar pattern is observed for subjective perception of health scores.

Table 2 presents a summary of the exogenous controls. The median age is 73 years, with 24 respondents over 95, including one aged 103. Women make up approximately 56.1% of the senior respondents. Regarding household composition, 55.2% are married couples, 32.8% live alone, 10.3% reside with children, and 1.7% belong to other household types.

Regarding the socio-economic characteristics, half of the respondents completed middle school or less. More than 60% report having no regular monthly income or work experience, prompting us to provide summary statistics for these variables excluding zero observations. Among those with a positive income, the median is 1.7 million won (approximately USD 1200 per month at the current exchange rate). Respondents with work experience have worked, on average, for 20 years.

Three percent of respondents report conflicts with their children in the past year, while seven percent report conflicts with their spouses. The technical median for these binary variables is zero, though this carries no meaningful interpretation. Half of the respondents express satisfaction with their current dwelling, corresponding to a score of 75%. The majority (9226 out of 10,078) perceive their environment as safe.

### 3.2. First-Stage Instrumented Regressions

As discussed above, in the first stage of the 2SLS estimation procedure, the endogenous determinants of life satisfaction are regressed on their corresponding instruments, which are specific to each endogenous determinant, along with a common set of exogenous controls that are also included in the first stage.

To assess the validity of the instruments, we employ the Hansen J test for overidentifying restrictions [41], which evaluates whether the instruments are uncorrelated with the error term and correctly excluded from the estimated equation. The test yields a chi-squared statistic of 3.47 with 4 degrees of freedom and a *p*-value of 0.483. Since the *p*-value is well above conventional significance levels, we fail to reject the null hypothesis, suggesting that the instruments are valid and that the endogeneity issue is appropriately addressed.

Table 3 reports coefficient estimates from regressions of the three endogenous variables on their respective instrument sets. The instrumented regressions indicate that social connections are stronger in rural areas and among individuals with a religion, internet access, a more adaptable attitude toward change, and a greater number of nearby friends. Physical activity is positively associated with infrastructure quality, while subjective health tends to be better in urban areas and among smokers but declines with higher alcohol consumption. Interestingly, better accessibility of government services is negatively associated with the level of physical activity.

Table 4 presents coefficient estimates from regressing endogenous determinants on exogenous controls. Our results imply that age negatively impacts social connections, physical activity, and subjective health, while education consistently boosts all three. Gender has little effect on physical activity or subjective health, but women tend to have stronger social connections. Married individuals enjoy better social ties and report higher subjective health, while higher income is linked to better health but less physical activity. Interestingly, satisfaction with one’s housing has a positive influence across all three indicators, and work experience fosters stronger social connections.

### 3.3. Endogeneity Tests

As discussed above, the three determinants of life satisfaction we employ are likely endogenous, as causality between these determinants and life satisfaction may run in both directions. In this section, we present formal tests for the endogeneity of social connections, physical activity, and subjective perception of health.

Table 5 presents the results of a residuals-based test based on Hausman [40]; see specification (3). The idea is to use predicted residuals from the instrumented regressions estimated in the previous section as independent regressors in the estimation of the second-stage regression, see specification (2) above. To save space, we only report coefficient estimates for the three predicted residuals.

The predicted residuals in specification (3) are statistically significant, and the joint *F*-test confirms the endogeneity of social connections, physical activity, and subjective health with respect to life satisfaction. These results support the use of instrumental variables within the 2SLS framework.

### 3.4. Second-Stage Regression Results

Table 6 reports the second-stage 2SLS results in (2) for the full sample and for separate rural and urban subsamples, incorporating the rural–urban dummy.

The key findings are as follows:

- Social connections, physical activity, and subjective health are positively associated with life satisfaction across all subsamples.
- Age, income, and years of work experience also exhibit consistently positive effects.
- Education, unexpectedly, shows a negative coefficient in all models.
- Dwelling satisfaction is positively linked to life satisfaction in rural areas but negatively associated in urban areas.

In the pooled model, dwelling satisfaction refers to an aggregate measure of satisfaction with one’s housing situation, combining data from both rural and urban areas. The pooled model does not differentiate between the two settings, so dwelling satisfaction is treated as a single variable that captures overall satisfaction across the entire sample.

As noted, the link between dwelling satisfaction and life satisfaction varies by region—positive in rural areas but negative in urban ones—indicating context-dependent effects masked in pooled models. Conflicts with spouses and children reduce life satisfaction across the board, with rural men and urban women reporting higher life satisfaction than their counterparts. These patterns underscore the need for context-specific policies, discussed in the next section.

**Figure 1:**
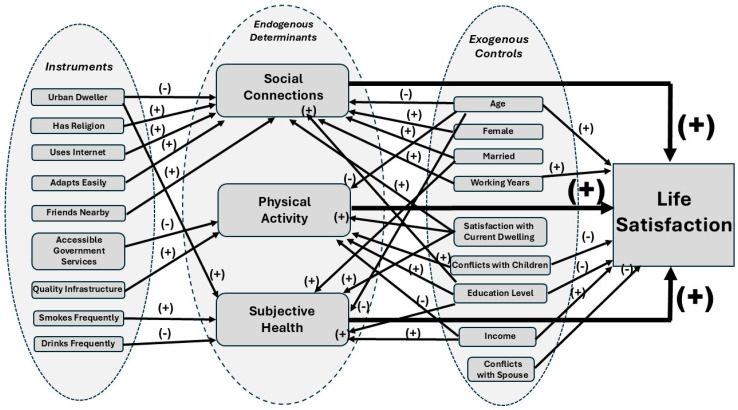
Conceptual framework of the determinants of life satisfaction.

The following Figure 1 summarizes the relationships we discovered between older Koreans’ life satisfaction, instrumental and endogenous determinants, and exogenous regressors.

## 4. Discussion

### 4.1. Endogenous Regressors and Instruments

We find that the urban–rural dummy variable is statistically significant as a determinant of the extent of social connectedness and the level of subjective health perception.

#### 4.1.1. Social Connectedness and Rural–Urban Divide

The negative link between social connectedness and the urban–rural dummy suggests that, contrary to common belief, urban areas may foster more fragmented, less personal social ties—possibly due to greater social isolation and more transactional relationships. These findings align with the Korean Social Life, Health and Aging Project (KSHAP, [42]), which reported higher depression scores among urban elderly (10.07) compared to rural elderly (5.82). They also support Park et al. [42], who found that social connectedness reduces depressive symptoms in older adults. Social ties may play a particularly crucial role for rural elderly, who often rely more on relationships to meet their needs, unlike urban elderly who may depend more on financial resources. Given that rural older adults typically maintain close, face-to-face relationships within tight-knit communities, these connections likely have a stronger effect on reducing psychological stress and enhancing life satisfaction.

Additionally, extensive access to digital technology in urban areas may reduce face-to-face interactions, potentially heightening social isolation. Stockwell et al. [43] found that UK older adults using the Internet or email once a week or month were less likely to be socially isolated than daily users. Our findings add nuance by showing that older individuals with better Internet access and greater adaptability to fast-changing informational environments report higher social connectedness. Thus, while technology may reduce in-person contact, its impact depends on how effectively it is used.

A pronounced socio-economic divide in urban areas may hinder social connection. While forming relationships online can reduce depression and loneliness and improve life satisfaction, many Koreans aged 65+ have low digital literacy, leading to information exclusion (Choi and Song [44]). Digital literacy is closely tied to social polarization, as disparities in economic, social, and cultural capital limit access. Older adults’ digital skills affect emotional wellbeing—depression, loneliness, anxiety, and self-esteem—which in turn influence life satisfaction. Thus, low digital literacy may deepen social isolation among older adults.

These findings are consistent with studies like Putnam [45], which highlight growing social isolation in urban areas marked by weaker ties and fragmentation. Although digital technologies could expand networks, Kraut et al. [46] show they may instead weaken social ties. In contrast, Coleman’s [47] social capital theory suggests that rural areas foster stronger, enduring personal relationships and communal support.

#### 4.1.2. Physical Activity and Infrastructure Quality

Our results indicate no significant urban–rural difference in promoting physical activity among older adults, diverging from studies like Contrady et al. [48] that emphasize rural advantages. Instead, we find that physical activity correlates positively with infrastructure quality—such as accessible sidewalks, parks, and recreational facilities—supporting Sallis et al. [49], who stress the role of infrastructure in supporting active aging, especially for those with mobility challenges.

Our finding that better access to government services, such as food delivery and government-run restaurants, is linked to lower physical activity is unexpected. It may suggest that improved services allow older adults to stay indoors, reducing the need for outdoor activity. This contrasts with studies like Pinheiro et al. [50], which typically associate better public services with higher physical activity levels.

#### 4.1.3. Subjective Health, Smoking, and Alcohol Consumption

Expectedly, we find that urban residents report higher subjective health, which correlates negatively with alcohol consumption. However, subjective health also correlates positively with smoking, which is counterintuitive given smoking’s known health risks. This may be explained by the Dunning–Kruger effect, where individuals with poor health behaviors overestimate their health, as noted in Jia et al. [51].

### 4.2. Effects of Endogenous Regressors and Exogenous Controls

Our findings align with existing research, showing a positive link between life satisfaction and factors like social connectedness, physical activity, and subjective health perception. The statistically significant positive coefficients in both the pooled and urban–rural samples suggest that these associations are robust across environments.

#### 4.2.1. Life Satisfaction and Age

In all three samples, we find a positive link between age and life satisfaction. This may be explained by Carstensen’s [52] socioemotional selectivity theory, which suggests that older adults prioritize emotional wellbeing and meaningful relationships. Alternatively, the U-shape theory of life satisfaction [53] attributes this to midlife crisis effects and a more positive outlook in older age.

Research on life satisfaction across age groups shows mixed results. Some studies suggest satisfaction increases with age, despite initial declines due to pressures like retirement or children’s marriages, as noted by Kim and Yoo [54]. Gender differences in life satisfaction vary, but urban elderly often report higher satisfaction than rural counterparts. Park [55] highlights that rural-dwelling women have the lowest life satisfaction, indicating a need for focused attention on their wellbeing.

Our findings show that while age negatively affects life satisfaction through social connections, physical activity, and subjective health, its direct effect on life satisfaction is positive.

#### 4.2.2. Life Satisfaction and Living Conditions

The positive association between dwelling satisfaction and life satisfaction in rural areas is expected, but the negative association observed in urban areas warrants further explanation. Rural older adults tend to report higher satisfaction with neighborhood relationships and the physical environment, while their urban counterparts are more satisfied with access to services. Choi et al. [56] also find that rural seniors exhibit stronger attachment to their homes. For rural residents, dwelling satisfaction may reflect emotional attachment, continuity, and close-knit community ties—all of which contribute positively to overall wellbeing. In contrast, although urban seniors benefit from better amenities and healthcare access, these advantages may not translate into higher life satisfaction. As Clary [57] suggests, stronger social comparison pressures in urban settings may lead to feelings of residential alienation or unmet lifestyle expectations, diminishing the psychological benefits of good housing conditions.

Moreover, while our primary focus is on social, behavioral, and health-related determinants, housing conditions warrant closer examination. Prior studies (Jeong, 2014 [36]; Park et al., 2022 [37]) have demonstrated that housing tenure—whether one owns or rents—can significantly influence older adults’ sense of security, autonomy, and overall life satisfaction. In the Korean context, where homeownership is strongly associated with stability and social standing, tenure type and housing satisfaction may interact with other life domains to shape wellbeing. Incorporating these variables into future models would offer a more comprehensive understanding of life satisfaction among older adults.

#### 4.2.3. Life Satisfaction and Educational Achievement

We find a surprisingly robust negative association between educational achievement and life satisfaction among older Korean adults in all three samples. In Korea, where education is highly valued, retirees may feel a sense of loss if their educational achievements do not lead to financial security or fulfillment. Despite 61.7% of the elderly having completed junior high school, their pride in Korea’s economic success may influence their outlook.

Higher education may lead to cognitive dissonance, where the idealized notion of educational achievement conflicts with post-retirement reality. In the Korean context, where education has long been associated with social status and economic mobility, older adults with higher education may experience a sense of unfulfilled expectations if their post-retirement life falls short of their earlier aspirations. This disconnect can lead to cognitive dissonance, particularly among those whose educational achievements did not yield lasting financial security or social recognition. This explanation appears to be especially plausible given the ongoing discourse about the need to reform the current retirement pension system in the country, as discussed, for example, in Pak [58].

#### 4.2.4. Other Determinants of Life Satisfaction

Conflicts with spouses and children generally reduce life satisfaction, while higher income and longer work histories are positively associated with life satisfaction, regardless of rural–urban differences.

Gender differences in life satisfaction vary by living environment. In urban areas, older women report higher satisfaction than men, while in rural areas, the opposite is true. This may be due to women benefiting from better access to services in urban areas, while rural areas may present a more male-oriented environment, as highlighted by Marmot [59].

## 5. Conclusions and Policy Implications

This study examines factors influencing life satisfaction among older adults in Korea, focusing on social connectedness, physical activity, and subjective health, with urban–rural differences shaping life satisfaction through these factors. Urban areas offer better infrastructure but tend to have fragmented social networks and higher isolation, while rural areas, despite lacking infrastructure, benefit from stronger social ties and support systems.

Our results highlight the importance of supportive environments for older adults in both urban and rural areas. The government should invest in policies that promote social connectedness in urban areas, such as community-building initiatives and spaces for face-to-face interactions.

Our findings suggest that accessible government services, like food delivery and public restaurants, may reduce physical activity. Policymakers thus should focus on programs that promote outdoor activities and social participation, preventing these services from fostering sedentary lifestyles.

The negative link between education and life satisfaction among older Koreans highlights the need for better support for well-educated retirees. Policymakers should consider programs that connect older adults with employment opportunities matching their educational background.

Our findings show that urban women report higher life satisfaction than men, while the opposite is true in rural areas. Policymakers should explore these gender differences and design interventions that address both gender and the urban–rural divide. These measures should be coordinated by local governments and community health centers, with a focus on empowering rural women and challenging traditional gender roles in rural leadership.

Korea is preparing for the full implementation of the Act on Integrated Support for Regional Care, which passed in April 2024 and will be enacted by 2026. This law shifts care provision from the central to local governments, creating a comprehensive system that integrates healthcare, long-term care, daily support, housing, and welfare. Local governments will need to assess care needs and develop tailored eldercare models. Identifying factors that influence life satisfaction among older adults is crucial for creating effective integrated care policies.

Our findings highlight the need for policies that prioritize social connectedness, inclusive infrastructure, and supportive services to enhance life satisfaction for all older adults, regardless of their living environment.

First, our analysis cautions against assuming uniform effects of housing quality or education across regions or cohorts. Policy interventions aimed at improving older adults’ wellbeing must be context-sensitive, addressing not only physical housing conditions but also psychological, emotional, and community-based dimensions. Second, eldercare and housing policies should recognize the symbolic and emotional meaning of ‘home’ for older adults, especially in rural areas. Third, the results highlight the need for more nuanced indicators of wellbeing that go beyond economic or structural measures.

For aging policy in Korea, several priorities emerge. In urban areas, investing in community infrastructure—such as senior hubs that combine social, educational, and physical activity programs—can help reduce isolation and promote healthy aging. In rural areas, where social cohesion is stronger, but service access is limited, mobile outreach and intergenerational programs can strengthen social capital while addressing unmet needs.

The inverse relationship observed between service accessibility and physical activity suggests the risk of encouraging sedentary lifestyles. To address this, programs like public meal services could be paired with outdoor or group activities. Gender-specific patterns in life satisfaction also call for tailored approaches: urban women may benefit from greater opportunities for engagement and leadership, while rural women may need support in navigating traditional role constraints.

Finally, with the upcoming implementation of Korea’s Integrated Care Act in 2026, local governments must ensure that care models align with the diverse social and health realities of their elderly populations. This will require region-specific data, inclusive planning, and participatory governance to ensure integrated services enhance, rather than compromise, wellbeing.

## Figures and Tables

**Table 1:** Life satisfaction, social connectedness, physical activity, and subjective perception of health.

	Median	Standard Deviation	Min	Max
**Life Satisfaction**	3	0.662	1	5
**Social Connectedness**	0.388	0.155	0	1
**Physical Activity**	0.286	0.385	0	1
**Subjective Perception of Health**	0.5	0.220	0	1

Note: the number of observations is 10,078 in all cases. Higher scores correspond to more desirable outcomes.

**Table 2:** Exogenous Controls: Age, Gender, and Marital Status.

	Mean	Median	Standard Deviation	Min	Max
**Age**	74.13	73	6.809	65	103
**Gender**	0.62	1	0.486	0	1
**Marital Status**	0.59	1	0.492	0	1
**Education**	0.477	0.5	0.172	0	1
Income *	165.63	150	150.68	15	3000
Working Years *	19.38	15	16.706	1	75
**Conflicts with Children**	0.027	NA	0.163	0	1
**Conflicts with Wife**	0.073	NA	0.261	0	1
**Satisfaction with Current Dwelling**	0.686	0.75	0.159	0	1
**Worrying about Crime**	0.013	NA	0.049	0	1

Note: the number of observations is 10,078 in all cases. Higher scores correspond to more desirable outcomes. * marks those variables for which summary statistics are given excluding the zero values. Gender: 0 if male, 1 if female. Marital status: 0 if no spouse; 1 if there is a spouse. Education level: 1 if illiterate; 2 if literate; 3 for elementary school; 4 for middle school; 5 for high school; 6 in case of a 2-year college degree; 7 in case of a 4-year university degree; 8 in case of a graduate degree, normalized to the range between zero and one. Income: monthly, 10,000 Korean won; satisfaction with current dwelling: 0 very dissatisfied; 1 dissatisfied; 2 fairly satisfied; 3 satisfied; 4 very satisfied, normalized to the range between zero and one.

**Table 3:** Effects of instruments on endogenous regressors.

	Social Connections	Physical Activity	Subjective Health
**Rural**–**Urban Dummy**	−0.014 (0.003) ***	0.004 (0.009)	0.017 (0.005) ***
**Religion Dummy**	0.020 (0.003) ***		
**Internet Dummy**	0.035 (0.003) ***		
**Adaptability Dummy**	0.095 (0.011) ***		
**Nearby Friends**	0.192 (0.004) ***		
**Government Services Accessibility**		−0.129 (0.037) ***	
**Infrastructure Quality**		0.367 (0.032) ***	
**Smoking Frequency**			0.011 (0.004) ***
**Alcohol Consumption Frequency**			−0.067 (0.009) ***

Note: robust standard errors are in parentheses. *** stands for a 1% significance level

**Table 4:** Effects of common exogenous controls on endogenous regressors.

	Social Connections	Physical Activity	Subjective Health
**Age**	−0.0004 (0.0002) *	−0.004 (0.001) ***	−0.004 (0.0006) ***
**Gender**	0.040 (0.003) ***	−0.002 (0.017)	−0.003 (0.012)
**Married Dummy**	0.015 (0.003) ***	0.004 (0.016)	0.066 (0.008) ***
**Conflicts with Spouse**	−0.0001 (0.005)	0.033 (0.031)	−0.018 (0.016)
**Conflicts with Children**	−0.010 (0.008)	0.094 (0.049) *	0.005 (0.025)
**Education level**	0.073 (0.010) ***	0.253 (0.055) ***	0.249 (0.028) ***
**Income level**	0.000 (0.000)	−0.0003 (0.00008) ***	0.0002 (0.00003) ***
**Working Years**	0.0007 (0.0001) ***	−0.0006 (0.0004)	0.0003 (0.0002)
**Satisfaction with Current Dwelling**	0.140 (0.009) ***	0.101 (0.044) **	0.192 (0.023) ***
**Criminal Environment Perception**	0.041 (0.028)	0.062 (0.225)	−0.110 (0.136)

Note: robust standard errors are in parentheses. *** stands for a 1% significance level, ** indicates a 5% significance level, and * indicates a 10% significance level.

**Table 5:** Residual-based test of endogenous regressors.

Dependent Variable: Life Satisfaction	Coefficients on Predicted Residuals/ *F*-Statistic
**Residuals from Social Connections**	−0.149 (0.087) *
**Residuals from Physical Activity**	−1.550 (0.137) ***
**Residuals from Subjective Health**	−0.793 (0.098) ***
***F*-statistic**	71.38 (0.000) ***

Note: robust standard errors are in parentheses. *** stands for a 1% significance level, and * indicates a 10% significance level.

**Table 6:** Second-stage estimation results.

Dependent Variable: Level of Life Satisfaction			
	Pooled Sample	Rural	Urban
**Predicted Values of the Endogenous Regressors**			
**Social Connections**	1.384 (0.100) ***	1.740 (0.237) ***	1.075 (0.113) ***
**Physical Activity**	2.012 (0.143) ***	1.511 (0.248) ***	2.562 (0.181) ***
**Subjective Health**	1.426 (0.098) ***	1.344 (0.173) ***	1.595 (0.118) ***
**Exogenous Controls**			
**Age**	0.010 (0.001) ***	0.011 (0.002) ***	0.010 (0.002) ***
**Gender**	−0.009 (0.015)	−0.065 (0.027) **	0.033 (0.018) *
**Married**	0.0006 (0.014)	0.031 (0.024)	−0.015 (0.017)
**Conflicts with Spouse**	−0.101 (0.023) ***	−0.094 (0.047) **	−0.088 (0.026) ***
**Conflicts with Children**	−0.096 (0.041) **	−0.153 (0.188) *	−0.032 (0.047)
**Education Level**	−0.497 (0.064) ***	−0.522 (0.123) ***	−0.496 (0.074) ***
**Income**	0.0006 (0.00008) ***	0.0003 (0.0001) **	0.0008 (0.0001) ***
**Years at Work**	0.005 (0.0005) ***	0.002 (0.0007) ***	0.008 (0.0009) ***
**Dwelling Satisfaction**	0.079 (0.062)	0.337 (0.086) ***	−0.173 (0.085) **
**Worrying about Crime**	−0.180 (0.122)	−0.182 (0.288)	−0.083 (0.133)

Note: robust standard errors are in parentheses. *** stands for a 1% significance level, ** indicates a 5% significance level, and * indicates a 10% significance level.

## Data Availability

This dataset is owned by the Korea Institute for Health and Social Affairs (KIHASA). In accordance with the law, KIHASA implements an open data policy for public data. Access to the data can be granted by the authors upon request, subject to review and approval by KIHASA.
